# Bacterial Cellulose-Modified Polyhydroxyalkanoates Scaffolds Promotes Bone Formation in Critical Size Calvarial Defects in Mice

**DOI:** 10.3390/ma13061433

**Published:** 2020-03-21

**Authors:** Ada Codreanu, Cornel Balta, Hildegard Herman, Coralia Cotoraci, Ciprian Valentin Mihali, Nicoleta Zurbau, Catalin Zaharia, Maria Rapa, Paul Stanescu, Ionut-Cristian Radu, Eugeniu Vasile, George Lupu, Bianca Galateanu, Anca Hermenean

**Affiliations:** 1 Faculty of Medicine, Vasile Goldis Western University of Arad, 94–96 Revolutiei Avenue, 310025 Arad, Romania; ada.codreanu@yahoo.com (A.C.); ccotoraci@yahoo.com (C.C.); mihaliciprian@yahoo.com (C.V.M.); dr.anghelnicoleta@yahoo.ro (N.Z.); 2 “Aurel Ardelean” Institute of Life Sciences, Vasile Goldis Western University of Arad, 86 Rebreanu Street, 310414 Arad, Romania; baltacornel@gmail.com (C.B.); hildegard.i.herman@gmail.com (H.H.); 3 Advanced Polymer Materials Group, University Politehnica of Bucharest, 060042 Bucharest, Romania; zaharia.catalin@gmail.com (C.Z.); paul.stanescu@upb.ro (P.S.); radu.ionucristian@gmail.com (I.-C.R.); 4 Faculty of Materials Science and Engineering, University Politehnica of Bucharest, 060042 Bucharest, Romania; rapa_m2002@yahoo.com; 5 Department of Oxide Materials Science & Engineering, University Politehnica of Bucharest, 011061 Bucharest, Romania; eugeniuvasile@yahoo.com; 6 Department of Anatomy, University of Medicine and Pharmacy Carol Davila, 050471 Bucharest, Romania; lupogeorge@yahoo.com; 7 Department of Biochemistry and Molecular Biology, University of Bucharest, 050471 Bucharest, Romania; bianca.galateanu@bio.unibuc.ro

**Keywords:** bacterial cellulose, polyhydroxyalkanoates, bone tissue engineering, in vivo tests

## Abstract

Bone regeneration is a claim challenge in addressing bone defects with large tissue deficits, that involves bone grafts to support the activity. In vitro biocompatibility of the bacterial cellulose-modified polyhydroxyalkanoates (PHB/BC) scaffolds and its osteogenic potential in critical-size mouse calvaria defects had been investigated. Bone promotion and mineralization were analyzed by biochemistry, histology/histomorphometry, X-ray analysis and immunofluorescence for highlighting osteogenesis markers. In summary, our results showed that PHB/BC scaffolds are able to support 3T3-L1 preadipocytes proliferation and had a positive effect on in vivo osteoblast differentiation, consequently inducing new bone formation after 20 weeks post-implantation. Thus, the newly developed PHB/BC scaffolds could turn out to be suitable biomaterials for the bone tissue engineering purpose.

## 1. Introduction

Bone regeneration is a major claim in addressing bone defects with large tissue deficits, that involves bone grafts to support the activity. The bone defects structure and function can be restored with autologous bone grafting, allogeneic grafting from another patient, xenografting from other species or artificial substitutes [1].

However, the last three listed alternatives are susceptible for an immune response, graft rejection, pathogen transmission and could lead to overall poor osseointegration. Therefore, autologous grafts exhibit the best clinical outcome, but it is limited by the donor sites quantities and size, which may not be appropriate for repair of large bone defects [2]. Together, these findings highlight the clinical needs for development of the new bone grafting materials and the demand to build up replacement materials that can be immediately processed for larger bone defects.

To date, natural biopolymers designed for bone regeneration have been one of the main aim of medical research field in the last ten years. Collagen, gelatin, chitosan and particularly cellulose were used in regenerative medicine because of the similar properties to the native tissue [3].

Bacterial cellulose (BC) is a natural biopolymer, free of lignin and hemicellulose, with high biodegradability and biocompatibility [4,5]. The excellent properties recommended BC for extensive use in regenerative medicine, such as vascular grafting, wound dressings, artificial skin and bone tissue engineering [4,5,6,7,8,9]. However, BC works only as a physical barrier against microbes, being hardly absorbed in the body and has no antibacterial properties [4]. Besides, BC dried nanofibrils are dense networks that can greatly decrease cell adhesion, infiltration and proliferation [10].

Recent results exhibit several materials based on bacterial cellulose, such as chitosan-BC [11], BC-silk fibroin (SF) [4], BC-hydroxyapatite (Hap) [12,13], BC-poly(3-hydroxybutyrate-co-3-hydroxyvalerate) (PHBHV) [14], BC-functionalized multiwalled carbon nanotubes (MWNTs) [15], BC-polymethacrylate nanocomposites [16], BC-montmorillonite (MMT) [17] designed for tissue engineering.

One of the most used bacterial-derived polymers, polyhydroxyalkanoates (PHAs), is giving promising results in bone regenerative medicine, because it possesses enhanced mechanical properties, improved biodegradability and favorable biocompatibility [18]. In our previous study, we reported the physico-chemical characterization and the in vitro biocompatibility of the natural materials based on BC and poly(3-hydroxybutyrate-co-3-hydroxyvalerate), as biomaterials for regenerative medicine purpose [14].

Therefore, based on our previous data and findings regarding osteoinduction, osteoconduction, osteointegration properties of BC [8], we aimed to extend biomedical applications of the bacterial cellulose—modified poly(3-hydroxybutyrate) scaffolds (PHB/BC) to bone tissue engineering. However, to date, the effect of PHB/BC scaffolds on bone defect healing has not been addressed. Our aim was to evaluate the in vitro biocompatibility and to investigate the osteogenic potential of the PHB/BC scaffolds in critical-size mouse calvaria defects. Bone promotion and growing, distribution and mineralization were analyzed by biochemistry, histology and X-ray, immunofluorescence for highlighting osteogenesis markers.

## 2. Materials and Methods

### 2.1. Materials

Powder bacterial poly(3-hydroxybutyrate) (PHB) kindly supplied by BIOMER, City, Krailling, Germany is characterized by 0.5–50 μm size dimensions and melting point of 174 °C determined by DSC (823^e^, Mettler Toledo, Greifensee, Switzerland). Bacterial cellulose (BC) was synthesized by The National Chemical-Pharmaceutical for Research and Development Institute (ICCF Bucharest, Romania). Tributyl citrate (TBC) supplied by Sigma-Aldrich Chemie GmbH, Taufkirchen, Germany as bioplasticizer, shows: assay > 97.0%, N20/D of 1.445 and molecular mass of 360.44 g/mol. Granular sodium chloride (NaCl) particles, with the size in the range 420–500 μm, were used as porogen. Phosphate buffered saline (PBS) solution (pH 7.4) was prepared in laboratory. Other reagents used are of analytical grade.

### 2.2. Methods

#### 2.2.1. Obtaining of Bacterial Cellulose

BC was produced by *A. xylinum* DSMZ-2004 (German Collection of Microorganisms and Cell Cultures, Braunschweig, Germany) incubated in a static culture media, consisting of 0.5% glucose, 2% glycerol, 0.5% citric acid and 0.2% ammonium sulfate The culture media were inoculated with 10% (v/v) A. xylinum DSMZ-2004 and sterilized by autoclaving. The incubation was performed at 30 °C, for 14 days. Then, the BC sheets was treated with 4% NaOH 1 N sol., at 30 °C for 2 days, washed with 0.02% sodium azide. The pH was decreased to 7.0 by washing with 1% acetic acid and distilled water. At the end, BC was dried and milled in grinder set at 1500 rpm to obtain the bacterial cellulose powder.

#### 2.2.2. Preparation of PHB/BC Scaffolds

PHB/BC scaffolds were obtained according to the salt leaching technique [19]. PHB and BC 1% and respectively 2% in content (wt.%) were loaded in a Brabender Plastograph and melt mixed at 180 °C for 6 min and rotor speed of 40 rpm. TBC plasticizer was added to each melted formulation, so that the ratio between PHB:TBC was kept constant at 80:20. Then NaCl particles were added into melted mixtures and the mixing is continued for about 2 min. The samples notation and compositions of the prepared specimens are showed in Table 1.

Before mixing, the PHB was oven dried at 60 °C overnight. Also, the BC was dried in oven until 105 °C, for 2 h. The melted materials were pressed into sheets with thickness of max. 100 µm and thin films by compression molding using a laboratory press (Polystat T200, COLLIN Lab & Pilot Solutions GmbH, Maitenbeth, Germany) in the following conditions: 185 °C and 200 bars for 5 min. Subsequently, the sheets and films were repeatedly washed with distilled water for leaching out the embedded salt. After the salt-leaching process, the macroporous scaffolds were dried under vacuum.

#### 2.2.3. Characterization of the Polymeric Scaffolds

Morphological analysis of the scaffold covered with a gold layer and examined by scanning electron microscopy (SEM). The samples were cut in nitrogen to highlights their cross-sectional morphology.

The sample tensile properties were determined according to EN ISO 527:2012. An INSTRON Universal Testing Machine (Instron 3382, 100 kN cell force, 825 University Ave Norwood, MA, USA) operated at a crosshead speed of 1 mm/min was used for testing the specimen dog-bone tensile bars. The analysis was made on five samples/each composition and reported as an average. The functional groups in polymeric materials were identified by infrared spectra on a 2000–104 FTLA spectrometer (ABB, Quebec, QC, Canada). Film samples were analyzed under attenuated total reflectance (ATR) using a Se-Zn crystal, at 25 °C, under transmittance mode. The spectra were recorded over the range of 2000 to 750 cm^−1^ with an accumulation of 22 scans, resolution of 4 cm^−1^, using air as background. PHB powder was used as control sample.

#### 2.2.4. In Vitro Biocompatibility Evaluation

3T3-L1 preadipocytes mouse cell line (CL-173TM, ATCC) was used to test the samples biocompatibility in terms of: (i) cells distribution on the materials surface (DAPI fluorescent staining of the cells nuclei) and (ii) cell viability and proliferation ability after direct contact with the materials (MTT spectrophotometric quantitative test).

##### In Vitro Cell Culture Model

3T3-L1 preadipocytes were propagated in vitro, in standard conditions of culture, in Dulbecco’s Modified Eagle’s Medium (DMEM) low glucose (Sigma-Aldrich, Saint Louis, MO, USA), supplemented with 3.5 g glucose (Sigma-Aldrich), 1.5 g NaHCO_3_ (Sigma-Aldrich), 1% antibiotic-antimicotic (ABAM) (Sigma-Aldrich) and 10% fetal bovine serum (FBS) (Gibco). The tested materials were sterilized by exposure to UV for 6 h. To perform the biocompatibility investigations, the sterile samples were seeded with 3T3-L1 cells at an initial cell density of 3 × 10^3^ cells/cm^2^.

##### DAPI Staining of the Cell Nuclei

To investigate the 3T3-L1 preadipocytes distribution on the materials surface, at 24 h and 5 days after contact, the cells were fixed for 20 min in 4% paraformaldehyde. After PBS washing, the samples were incubated for 1 h in 2% bovine serum albumin (BSA) and Triton ×100 solution for cell membrane permeabilization. The nuclei were stained with 4′,6-diamidino-2-fenilindol (DAPI) by 15 min. incubation in dark. The blue fluorescence of the labeled nuclei was observed using an inverted microscope Olympus IX71 with fluorescence modulus (Miami, FL, USA). The images were captured using the CellF Software (Miami, FL, USA).

##### MTT Assay

Cell viability and proliferation potential of 3T3-L1 preadipocytes in contact with the materials were evaluated using the spectrophotometric MTT assay. Thus, the medium was replaced with 1 mg/mL MTT solution (Sigma-Aldrich) after 24 h and 5 days. After 4 h, the produced formazan crystals were solubilized in Dimethyl sulfoxide DMSO for 30 min. The optic density analyzed at 550 nm, using a TECAN spectrophotometer (Tecan Group Ltd. Mannedorf, Switzerland).

#### 2.2.5. In Vivo Osteogenic Potential Evaluation

##### Animal model and Surgical Procedure

**Figure 1:**
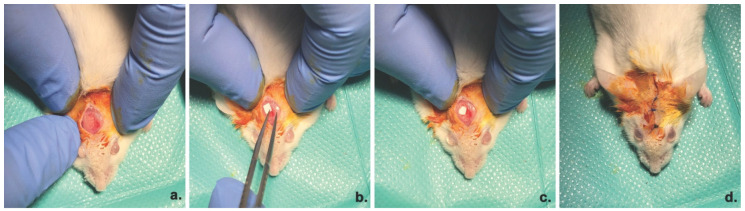
Surgical procedure. (**a**) 5-mm critical size calvaria defect (**b**,**c**) Placement of the PHB/BC scaffold (**d**) Closure of the surgical site.

Adult CD1 mice were used for the experimental protocol. The experiment was previously approved by the Ethics Committee for Research of the Vasile Goldis Western University of Arad. The critical bone defect surgery was performed under anesthesia by intraperitoneal (i.p.) administration of ketamine/xylazine. The 5 mm defects were prepared using a drill, under constant saline solution irrigation, as previously described [20]. The periosteum covered the defect site and the incisions were closed (Figure 1).

No mice died during surgery or post-surgical interval. Mice were assigned to the 4 experimental groups: Group 1: calvaria defect; Group 2 PHB:TBC scaffolds implanted; Group 3, (PHB:TBC)_BC1% scaffolds implanted; Group 4, (PHB:TBC)_BC2% scaffolds implanted; The scaffolds were sterilized by ultraviolet radiation (UV) for 30 min.

The animals were euthanatized after 3 days, four and twenty weeks (n = 10/time-interval/each group), using an overdose of anesthetic and the implants were extracted for further evaluation.

##### ALP Activity

Blood samples were collected into sterile containers by cardiac puncture. Alkaline phosphatase (ALP) serum concentration was measured by using a Chemistry Analyzer (Mindray BS-120, Shenzhen, China).

##### Radiographic Analysis

Radiograph images of the sites were taken by an in vivo imaging system (XTREME, Carestream, Rochester, NY, USA), following initial calibration. Each image received the same settings during the acquisition, as following: 1,200 sec. exposure time, 2.00 f-stop, 72.00 mm FOV, 2473 ppi vertical resolution, 2473 ppi horizontal resolution, 45 KVp X-ray energy, −60 °C temperature of the acquisition video camera CCD.

##### Histology and Morphometry Analysis

The calvaria new tissue, together with the surrounding soft and bony tissues were fixed in 4% paraformaldehyde for 3 days. Decalcification was performed in Biodec R (Bio-Optica, Milan, Italy) for 5 days. After dehydration and clarification, samples were included in paraffin. Histological sections of 5 μm thickness were cut and the slides were stained with Goldner’s Masson Trichrome (Bio-Optica). The examination was performed under an optical microscope (Olympus BX43, Miami, FL, USA).

The area of newly formed bone was measured as a calculated as a percentage reported to the total defect area and, as previously shown [20].

##### Immunofluorescence

The sections were deparaffinized, rehydrated and treated with sodium citrate buffer (pH 6.0) for antigen retrieval. After bovine serum albumin (BSA) blocking, a rabbit polyclonal Osterix antibody (Santa Cruz, sc-22536-R) in 1% BSA (4 °C overnight; 1:50 dilution) was added. Next, the samples were exposed to Alexa Fluor 488 (Cambridge, UK) goat anti-rabbit secondary antibody (1:1000) for 1 h in dark at room temperature. Nuclei were stained with DAPI. Slides were mounted and analyzed under a Leica TCS SP8 confocal microscope (Wetzlar, Germany).

#### 2.2.6. Statistical Analysis

The statistically processing was done with GraphPad Prism 3.03 software (GraphPad Software, Inc., La Jolla, CA, USA), using one-way analysis of variance, followed by a Bonferroni test. *p* < 0.05. was considered a statistically significant difference.

## 3. Results

### 3.1. Characterization of the PHB/BC Scaffolds

#### 3.1.1. Morphological Investigation

**Figure 2:**
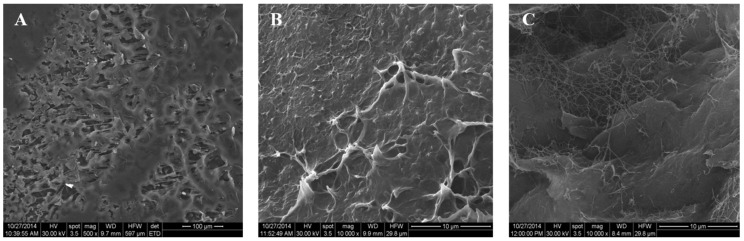
SEM microphotographs of PHB/BC scaffolds compared with plasticized PHB. (**A**) plasticized PHB; (**B**) (PHB:TBC)_BC1; (**C**) (PHB:TBC)_BC2.

The morphology of the prepared PHB and PHB/BC scaffolds was investigated by SEM (Figure 2). From Figure 2B,C it is observed that the PHB/BC scaffolds have interphasial adhesion between matrix and bacterial cellulose with small interconnected pores ranged between of 5–50 μm. It is known that the macropores are needed to allow the cell spreading of the material’s surface and core, proliferation and the blood growth vessels inside the network of pores [21,22,23].

#### 3.1.2. Fourier Transform Infrared Spectroscopy in Attenuated Total Reflectance mode (ATR-FTIR) Investigation

**Figure 3:**
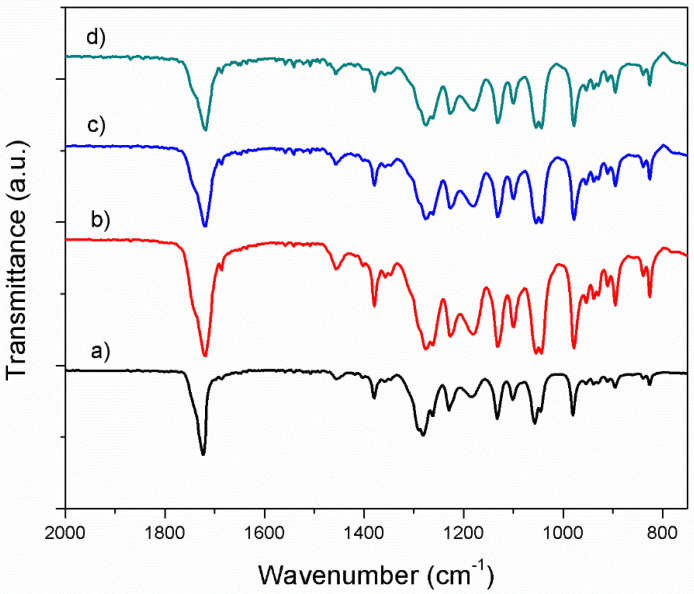
FTIR spectra of neat PHB and PHB blends (**a**) Neat PHB; (**b**) PHB:TBC; (**c**) (PHB:TBC)_BC1; (**d**) (PHB/:TBC)_BC2.

The spectral characteristics of neat PHB and its derivatives and blends with bacterial cellulose are shown in Figure 3. The main characteristic bands of neat PHB are ascribed to following frequencies: 895 cm^−1^ (the coupling of C–C backbone stretching with the crystalline C–O–C vibration bands), 980 cm^−1^ (the coupling of C–C backbone stretching with the CH_3_ stretching vibration), 1043 cm^−1^ (C-O bending), 1057 cm^−1^ (C–O–C stretching of crystalline part), 1032 cm^−1^, 1182 cm^−1^ (amorphous state of C–O–C stretching band), 1724 cm^−1^ (C=O stretching of crystalline part) and 1380 cm^−1^ (symmetric CH_3_ group deformation), very similar with those found in literature [24]. Together with the adding of plasticizer into neat PHB, it is observed that characteristic bands shifted at lower frequency (978 cm^−1^, 1055 cm^−1^, 1180 cm^−1^, 1720 cm^−1^ and 1718 cm^−1^) in comparison with neat PHB, due to the physical interactions between components [25]. The main characteristic bands of plasticized PHB are also found in PHB/BC materials, with some small shifts of their wave numbers. Therefore, carbonyl band shifts from 1720 cm^−1^ for plasticized PHB to 1718 cm^−1^ for PHB/BC scaffolds, as a result of the local molecular environment change during crystallization [26]. However, it is observed that the absorption bands intensity at 1718 cm^−1^ (C=O stretching) decreases with increasing the BC content which might be a hint of chemical degradation.

The crystallinity index (CI) was quantitatively evaluated by FTIR spectra as a ratio of the intensity heights of the reference band of CH_3_ (1380 cm^−1^) which is dull to the crystallinity range [27] to the amorphous band (1185 cm^−1^) [24] and is listed in Table 2.

As expected, for material with 2% bacterial cellulose, we registered a sharp decrease of crystallinity index of blends. This result shown that the crystalline fraction decreased with the increase of BC content with respect to neat PHB and PHB/TBC samples.

#### 3.1.3. Mechanical Evaluation of the Composite Samples

As expected, the tensile strength and elastic modulus of PHB/BC scaffolds increased with the introduction of bacterial cellulose into PHB polymer matrix–Table 3. Cellulose natural material, due to its renewability, biodegradability, high strength and stiffness, is considered suitable reinforcing agent for different polymeric materials towards various applications. The elastic modulus of a (bio)material plays an important role in controlling cell behaviour. Human tissue has different rigidities according to specific cell types and structural arrangement. Bone tissue has very high rigidity of around 5–10 GPa. The modulus of elasticity of PHB/TBC and PHB/BC samples ranges between 1250–1400 MPa (1.2–1.4 GPa) which is in the near range of the bone tissue values. By the adjustment of bacterial cellulose content, in vitro degradation together with desired mechanical properties of PHB /BC scaffolds could be promising candidates for bone tissue engineering.

### 3.2. In Vitro Biocompatibility of PHB/BC Scaffolds

#### 3.2.1. 3T3-L1 Cells Distribution on the Materials Surface

**Figure 4:**
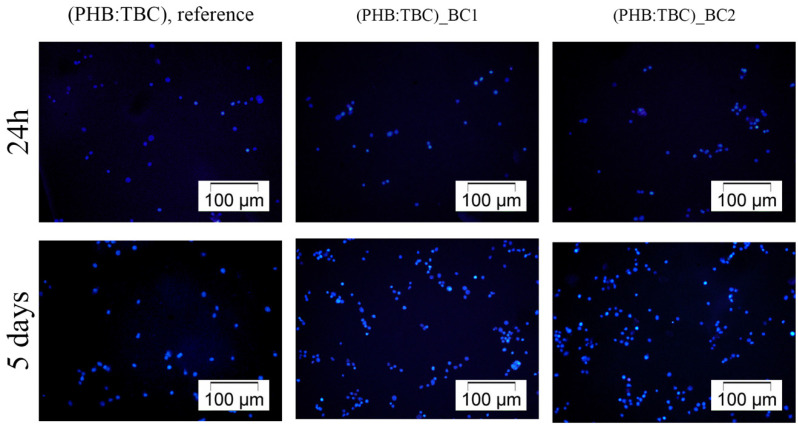
Fluorescence microscopy micrographs of DAPI stained nuclei (blue) in 3T3-L1 preadipocytes cultivated for 24 h and 5 days on the scaffolds surface. Scale 100 μm.

Fluorescence microscopy images captured after DAPI staining of the cell nuclei revealed that the 3T3-L1 cells were present on the tested materials surfaces (Figure 4) after 24 h and 5 days. Moreover, we observed an increased number of blue stained nuclei on the surface of the samples after 5 days post seeding as compared with 24 h of incubation, probably due to cells proliferation.

#### 3.2.2. 3T3-L1 Preadipocytes Viability and Proliferation Potential

**Figure 5:**
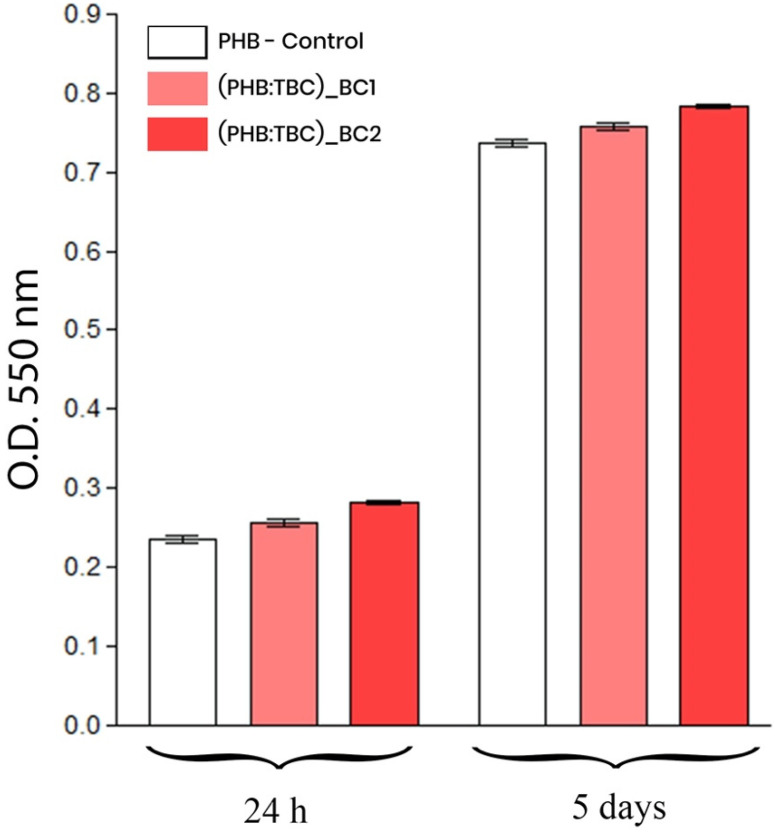
Graphic representation of the spectrophotometric data obtained by the MTT assay revealing the 3T3-L1 preadipocytes viability and proliferation potential after 24 h and 5 days.

All the above observations were confirmed by the spectrophotometric quantification of the cell viability at 24 h and 5 days post culturing as well as their proliferation potential during 5 days of culture. The quantitative data obtained after performing the MTT assay were graphically represented in Figure 5.

As shown in Figure 5, the 3T3-L1 mouse preadipocytes survived and proliferated 5 days on the tested materials surfaces. No differences between the materials were identified with this respect.

### 3.3. In Vivo Osteogenic Potential of BC/PHB Scaffolds

#### 3.3.1. Alkaline Phosphatase (ALP) Activity

**Figure 6:**
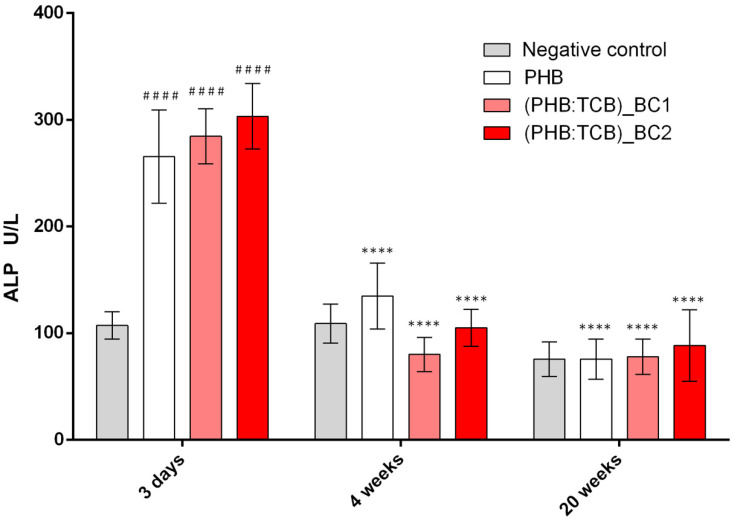
ALP activity in mice calvaria defects implanted with PHB/BC scaffolds after 3 days, 4 weeks and 20 weeks post-implantation. ### *p* < 0.0001 scaffods vs negative control (calvaria defect) at 3 days; **** *p* < 0.0001 3 days vs. 4 weeks (for each scaffold) si **** *p* < 0.0001 3 days vs. 20 weeks (for each scaffold).

As shown in Figure 6, the ALP level was significantly increased after 3 days, for implanted groups compared to the negative control (*p* < 0.001), the highest concentration registering for (PHB:TBC)_BC2. Next, the ALP decreased drastically to 4 weeks, respectively 20 weeks post-implantation.

#### 3.3.2. X-ray Analysis

**Figure 7:**
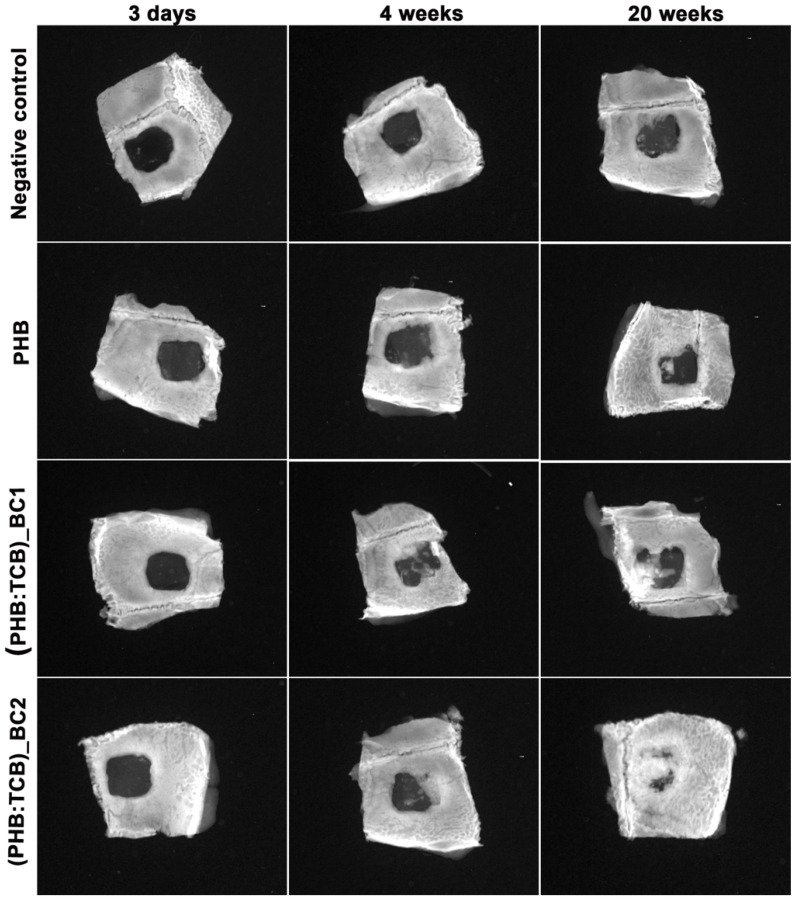
Radiographs of the in vivo bone samples taken after 3 days, 4 and 20 weeks of BC/PHB scaffold implantation. Radiological analysis showing gradual increase in bone formation in BC/PHB matrix compared to PHB matrix. Magnification ×2.5.

Mineralization of the new bone started to make progress at week 4, extending from the edges of the defect to the center. However, the radio-opacity in the mid region of the defect was low, showing less mineralization at this time-interval. The ossification is advanced at 20 weeks, due to deposition and in growth of the new bone and more advanced for (PHB:TBC)_BC2 (Figure 7).

#### 3.3.3. Histology and Histomorphometry Analysis

Histological findings of the calvaria defects at 20 weeks post-implantation showed that the bone defects filled with PHB/BC scaffolds, had superior recovery pattern compared to PHB alone group.

**Figure 8:**
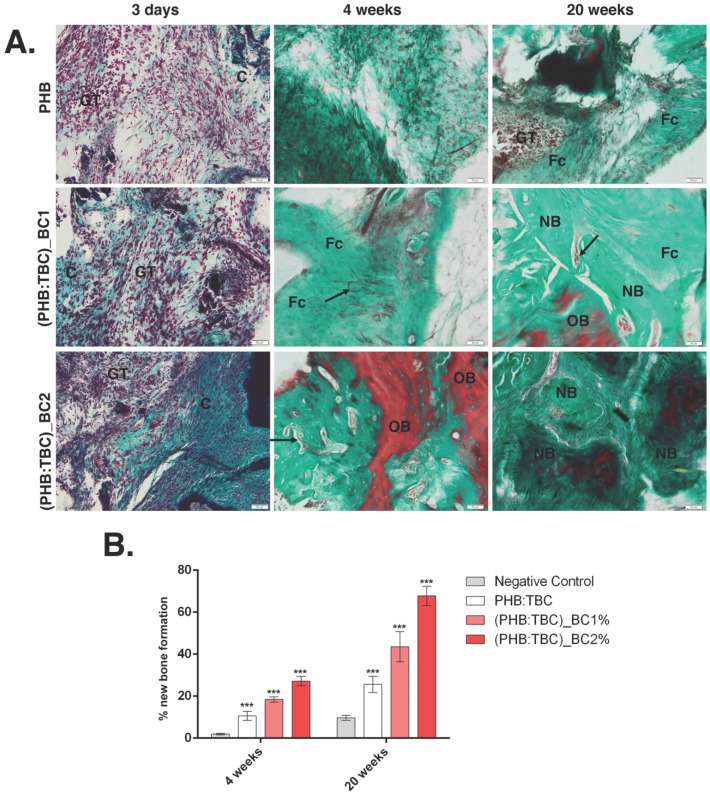
Histology and histomorphometry of the PHB/BC scaffolds after 3 days, 4 and 20 weeks post-implantation (**A**) Histology of the PHB, (PHB:TBC)_BC1 and (PHB:TBC)_BC2 after 3 days, 4 and 20 weeks of PHB/BC scaffolds implantation; symbols: scaffold (Sc); granulation tissue (GT); fibroconnective tissue (Fc); capillary (arrow); new bone (NB); old bone (OB); Masson Goldner trichrome stain. Scale 50 μm. (**B**) Histomorphometry showing bone recovery in calvaria defects implanted with CHB/BCs scaffolds after 4 and 20 weeks post-implantation, compared to negative control (only defect).

PHB/BC group led to an increased number of cells belonging to granulation tissue (GT) infiltrated from the edge to the center, 3 days post-implantation (Figure 8A). Fibroconnective tissue and vasculogenesis were increased in PHB/BC groups compared control at week 4, showing that PHB/BC scaffolds supported the restoration of the injured bone. The newly formed bone advanced from the edges toward the center.

At week 20, transition to mature bone tissue was observed. At this time-point, formation of new mature bone is obviously shown using Masson Goldner trichrome stain.

Histomorphometry revealed a significant difference between (PHB:TBC)_BCs groups and PHB alone, at 4 weeks, and 20 weeks respectively (*p* < 0.001) (Figure 8B).

#### 3.3.4. Osteogenic Differentiation of PHB/BC on Scaffolds

Osterix (OSX) is indispensable for osteoblast differentiation, proliferation and bone promotion [28,29]. Immunofluorescence positivity was found at the site of the implanted materials in week 4, raised gradually for PHB/BC scaffolds.

## 4. Discussion

For bone regenerative applications, important features are essential for biomaterials to heal large defects, as: biocompatibility and low inflammatory response, biodegradability and adapting the mechanical characteristics of the implant to the defect site [1].

PHAs with short chains, such as PHB with high mechanical strength, provided rapid proliferation of osteoblasts [30] and no chronic inflammatory reaction up to 12 months post-implantation [31]. Similar, HA reinforced PHBV had minimum inflammatory response and increased of mineralization level [32].

Bacterial cellulose has been studied for its beneficial properties useful for bone regenerative medicine, as: biocompatibility and promoting of cell’s interaction and tissue development, based on the cell adhesion, proliferation and ability to integrate well within the bone defect [33]; proper mechanical properties, due to the uniformly and high aspect ratio, cross-linked structure with ultrafine fibers and tensile strength ranged between 216 MPa and 260 MPa [7]; microporosity which could be controlled by the inoculum volume, culturing time and the drying method of the cellulose [34].

The scaffolds biocompatibility depends on the cell behavior after direct contact, and especially to the adhesion ability on their surface. Our in vitro results showed the ability of BC to promote cell adhesion to the PHB scaffolds, and to promote cell growth and proliferation, while regenerated bacterial cellulose/microfilaments of bacterial cellulose provided good cytocompatibility on a cell viability assay [35].

The aim of the in vivo study was to use previously described bio-patterning technology of both materials for bone tissue engineering [8] in order to investigate their application as potential “co-therapies” for healing critical-size calvaria defects. We tested the hypothesis that PHB/BC co-delivery will ensure the enhancing of the mechanical properties, a controllable biodegradability and biocompatibility in vitro, promoting the osteogenesis in vivo, in a mouse critical-size mouse calvaria model.

In this study, an in vivo study on the capacity of the PHB/BC to close a critical-sized calvaria defect was performed. A critical size defect is the smallest intraosseous defect which does not close spontaneously [36]. The calvaria-critical size defect is a common model for evaluating the osteogenic potential of the new materials, and where, without any assistance, the defect will fill with a fibrous connective tissue rather than with bone [37].

**Figure 9:**
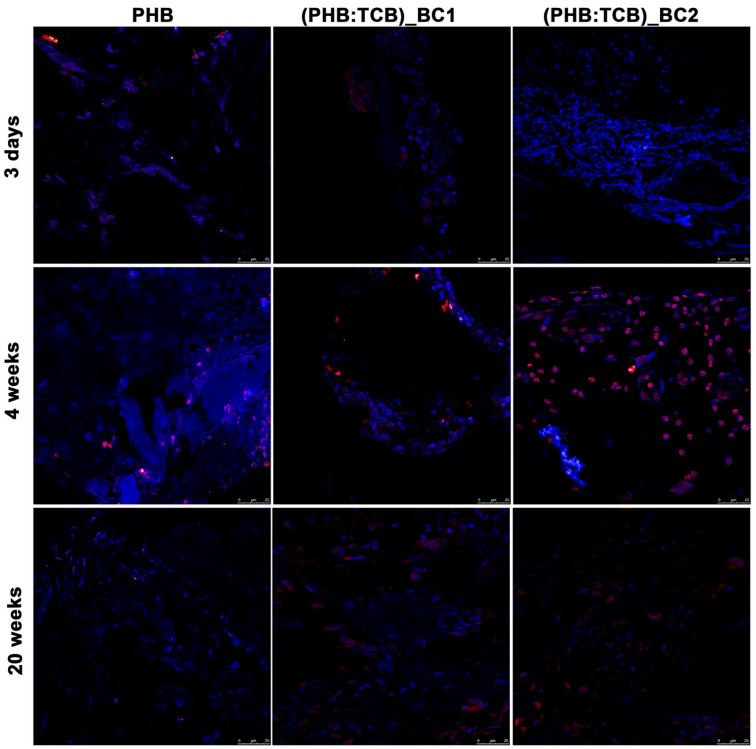
Immunofluorescent detection of osterix (OSX) in the repaired calvaria (in red) after 3 days, 4 and 20 weeks of PHB/BC scaffolds implantation. Cell nuclei were stained with DAPI (in blue). ×63 oil-immersion objective.

The tissue response to the implanted PHB/BC materials and the defect bone regeneration efficacy, was evaluated by histological analysis on the implant area (shown in Figure 9). The results of the present study showed that by adding of 2 wt.% BC to PHB significantly enhanced bone matrix production and mineralization at 20 weeks, when compared to 1 wt.% BC and PHB scaffolds, respectively. In agreement with other findings [38,39], histological sections demonstrated extensive bone formation within the edge to center of the defects and bridging when BC is present, being extra supported by the radiographic images. Furthermore, PHB scaffolds alone exhibit less bone formation, separated by a fibrous tissue layer. The mode of healing from the PHB alone and the PHB/BC scaffolds seemed to vary as histological/histomorphometry and X-ray evaluations, showed only peripheral healing for the non-BC scaffolds and extended regeneration throughout the defect site when BC was added, with increasing concentration. The addition of BC to the PHB biomaterials not only improved mechanical properties, but, according to high crystalline nature and lack of the enzymes which could break beta (1–4) glycosidic linkage of bacterial cellulose in the tissue, giving a progressive biodegradability to the scaffold [8]. This advantage has made it a suitable biomaterial for promoting bone remodeling. According to Nair and Laurencin [40], a good biodegradable biomaterial should degrade in time to adjust the healing process. In line with this statement, it was found that BC implanted subcutaneously in rats has degraded after 12 weeks [41,42].

The activities of progenitor cells to osteoblasts can be regulated during differentiation by different signaling pathways. The regulation is mediated by bone morphogenetic proteins BMPs and transcription factors such as Runx2 and Osterix [43,44]. Osterix is needed for the osteoblast differentiation [44,45] and regulates the expression of the main osteogenic markers, including alkaline phosphatase (ALP), Runx2, osteonectin, osteopontin and osteocalcin [28,46]. The strong OSX immunopositivity of the PHB/BC scaffolds after 4 weeks post-implantation is an indicator of osteogenesis enhancement mediated by the presence of scaffolds reinforced with bacterial cellulose.

ALP is one of the early enzymes expressed during osteogenesis, displayed on the outer surface of cells, and promotes mineralization via the release of inorganic phosphate. The process is initiated by the calcium and inorganic phosphate bioaccumulation, followed by crystal growth, mainly in the form of crystalline hydroxyapatite [47]. In our study, osteogenic activity of PHB/BC scaffolds was indicated by ALP activity and histological staining, which was much more highlighted than as for PHB alone or negative control. These results suggest that adding BC to PHB scaffolds, facilitated the differentiation of osteoprogenitor cells to an osteogenic cells, in the early stages. Our previous results also demonstrated that early stages of osteogenesis in vivo and osteoblast differentiation in vitro are associated with ALP enhanced activity [20].

## 5. Conclusions

The results of this study showed the following:

1. PHB/BC scaffolds are able to support 3T3-L1 preadipocytes viability and proliferation, with no signs of apparent toxicity and a good biocompatibility;
2. BC reinforced PHB had an enhanced effects on osteoblast differentiation in vivo, based on the increased OSX expression and enhanced ALP activity in the first 4 weeks post-implantation;
3. The X-ray and histology/histomorphometry analysis of the in vivo data showed that BC reinforced PHB induces new bone formation.

Thus, the newly developed PHB/BC scaffolds could turn out to be suitable biomaterials for the bone tissue engineering purpose.

## Figures and Tables

**Table 1:** Notations and composition of samples obtained by melt mixing technique.

Sample	PHB (wt.%)	TBC (wt.%)	BC (wt.%)	NaCl (wt.%)
Neat PHB	100	−	−	−
PHB:TBC	80	20	−	−
(PHB:TBC)_BC1	76.8	19.2	1	3
(PHB:TBC)_BC2	76	19	2	3

**Table 2:** Crystallinity index (CI).

Sample	A1380 cm^−1^/A1185 cm^−1^
Neat PHB	1.08
PHB:TBC	0.89
(PHB:TBC)_BC1	0.75
(PHB:TBC)_BC2	0.59

(A1380 cm^−1^/A1185 cm^−1^) from FTIR.

**Table 3:** Tensile properties for PHB and PHB/BC scaffolds.

Sample	Tensile Strength (MPa)	Elongation at Break (%)	Young Modulus (MPa)
PHB:TBC	9 ± 0.7	3 ± 0.2	1250 ± 98
PHB:TBC_BC1	12 ± 0.9	1.9 ± 0.4	1310 ± 75
PHB:TBC_BC2	15 ± 1.0	1.5 ± 0.3	1400 ± 101
