# Correction: Codreanu et al. Bacterial Cellulose-Modified Polyhydroxyalkanoates Scaffolds Promotes Bone Formation in Critical Size Calvarial Defects in Mice. *Materials* 2020, *13*, 1433

**DOI:** 10.3390/ma18184361

**Published:** 2025-09-18

**Authors:** Ada Codreanu, Cornel Balta, Hildegard Herman, Coralia Cotoraci, Ciprian Valentin Mihali, Nicoleta Zurbau, Catalin Zaharia, Maria Rapa, Paul Stanescu, Ionut-Cristian Radu, Eugeniu Vasile, George Lupu, Bianca Galateanu, Anca Hermenean

**Affiliations:** 1 Faculty of Medicine, Vasile Goldis Western University of Arad, 94–96 Revolutiei Avenue, 310025 Arad, Romania; ada.codreanu@yahoo.com (A.C.); ccotoraci@yahoo.com (C.C.); mihaliciprian@yahoo.com (C.V.M.); dr.anghelnicoleta@yahoo.ro (N.Z.); 2 “Aurel Ardelean” Institute of Life Sciences, Vasile Goldis Western University of Arad, 86 Rebreanu Street, 310414 Arad, Romania; baltacornel@gmail.com (C.B.); hildegard.i.herman@gmail.com (H.H.); 3 Advanced Polymer Materials Group, University Politehnica of Bucharest, 060042 Bucharest, Romania; zaharia.catalin@gmail.com (C.Z.); paul.stanescu@upb.ro (P.S.); radu.ionucristian@gmail.com (I.-C.R.); 4 Faculty of Materials Science and Engineering, University Politehnica of Bucharest, 060042 Bucharest, Romania; rapa_m2002@yahoo.com; 5 Department of Oxide Materials Science & Engineering, University Politehnica of Bucharest, 011061 Bucharest, Romania; eugeniuvasile@yahoo.com; 6 Department of Anatomy, University of Medicine and Pharmacy Carol Davila, 050471 Bucharest, Romania; lupogeorge@yahoo.com; 7 Department of Biochemistry and Molecular Biology, University of Bucharest, 050471 Bucharest, Romania; bianca.galateanu@bio.unibuc.ro

In the original publication [1], there was a mistake in Figure 9. The image corresponding to the condition “3 days (PHB:TCB)_BC1” has been updated, and the corrected Figure 9 appears below. The authors state that the scientific conclusions are unaffected. This correction was approved by the Academic Editor. The original publication has also been updated.

**Figure 9:**
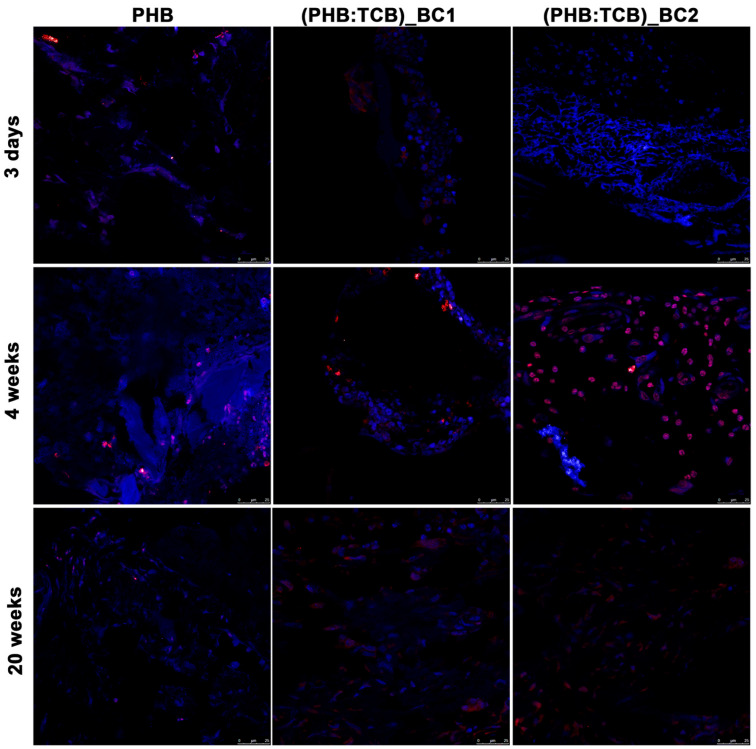
Immunofluorescent detection of osterix (OSX) in the repaired calvaria (in red) after 3 days, 4 and 20 weeks of PHB/BC scaffolds implantation. Cell nuclei were stained with DAPI (in blue). ×63 oil-immersion objective.
